# Bis(2,6-dihy­droxy­benzoato-κ^2^ *O* ^1^ *,O* ^1′^)(nitrato-κ^2^ *O*,*O*′)bis­(1,10-phenanthroline-κ^2^ *N*,*N*′)samarium(III)

**DOI:** 10.1107/S1600536810047136

**Published:** 2010-11-20

**Authors:** Guobo Hu, Chiya Wang, Dingfeng Jin, Hongxiao Jin

**Affiliations:** a College of Materials Science and Engineering, China Jiliang University, Hangzhou 310018, People’s Republic of China

## Abstract

The title mononuclear complex, [Sm(C_7_H_5_O_3_)_2_(NO_3_)(C_12_H_8_N_2_)_2_], is isostructural with that of other lanthanides. The Sm atom is in a pseudo-bicapped square-anti­prismatic geometry, formed by four N atoms from two chelating 1,10-phenanthroline (phen) ligands and by six O atoms, four from two 2,6-dihy­droxy­benzoate (DHB) ligands and the other two from a nitrate anion. π–π stacking inter­actions between phen and DHB ligands [centroid–centroid distance = 3.528 (4) and 3.812 (3) Å], and phen and phen ligands [face-to-face separation = 3.420 (10) Å] of adjacent complexes stabilize the crystal structure. Intra­molecular O—H⋯O hydrogen bonds are observed in the DHB ligands.

## Related literature

For background and details of a related structure, see: Zheng *et al.* (2010 ▶).
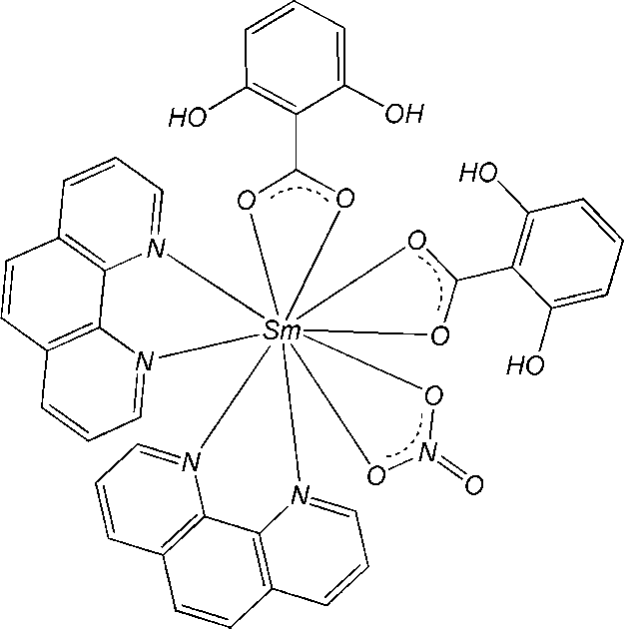


## Experimental

### 

#### Crystal data

[Sm(C_7_H_8_O_3_)_2_(NO_3_)(C_12_H_8_N_2_)_2_]
*M* *_r_* = 878.99
Monoclinic, 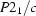

*a* = 11.2022 (3) Å
*b* = 26.7672 (7) Å
*c* = 14.3326 (5) Å
β = 127.635 (2)°
*V* = 3403.4 (2) Å^3^
*Z* = 4
Cu *K*α radiation
μ = 13.59 mm^−1^
*T* = 298 K
0.40 × 0.35 × 0.33 mm

#### Data collection

Oxford Diffraction Gemini S Ultra diffractometer
Absorption correction: multi-scan [*ABSPACK* in *CrysAlis PRO RED* (Oxford Diffraction, 2006 ▶)] *T*_min_ = 0.074, *T*_max_ = 0.094
11856 measured reflections
6027 independent reflections
5456 reflections with *I* > 2σ(*I*)
*R*_int_ = 0.045

#### Refinement

*R*[*F* ^2^ > 2σ(*F* ^2^)] = 0.048
*wR*(*F* ^2^) = 0.122
*S* = 1.06
6027 reflections
496 parameters
H-atom parameters constrained
Δρ_max_ = 1.36 e Å^−3^
Δρ_min_ = −2.76 e Å^−3^

### 

Data collection: *CrysAlis PRO CCD* (Oxford Diffraction, 2006 ▶); cell refinement: *CrysAlis PRO CCD*; data reduction: *CrysAlis PRO RED* (Oxford Diffraction, 2006 ▶); program(s) used to solve structure: *SHELXS97* (Sheldrick, 2008 ▶); program(s) used to refine structure: *SHELXL97* (Sheldrick, 2008 ▶); molecular graphics: *DIAMOND* (Brandenburg & Berndt, 1999 ▶); software used to prepare material for publication: *SHELXL97*.

## Supplementary Material

Crystal structure: contains datablocks I, global. DOI: 10.1107/S1600536810047136/su2228sup1.cif

Structure factors: contains datablocks I. DOI: 10.1107/S1600536810047136/su2228Isup2.hkl

Additional supplementary materials:  crystallographic information; 3D view; checkCIF report

## Figures and Tables

**Table 1:** Hydrogen-bond geometry (Å, °)

*D*—H⋯*A*	*D*—H	H⋯*A*	*D*⋯*A*	*D*—H⋯*A*
O4—H31⋯O2	0.82	1.87	2.594 (5)	147
O3—H27⋯O1	0.82	1.83	2.562 (6)	149
O8—H38⋯O6	0.82	1.85	2.578 (6)	147
O7—H34⋯O5	0.82	1.86	2.589 (5)	147

**Fig. 1.**
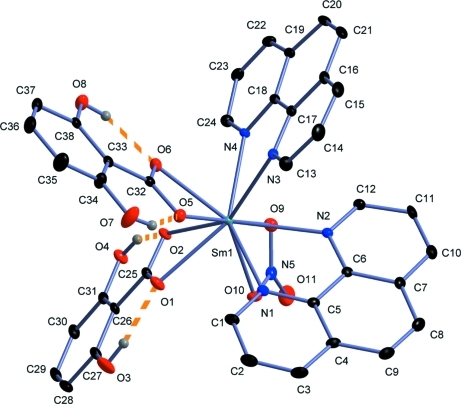
The molecular structure of title compound. Displacement ellipsoids are drawn at the 15% probablility level and H atoms are shown as small spheres of arbitraty radii. Some H atoms are omitted for clarity. Light orange lines show the intramolecular hydrogen bonds (see Table 1 for details).

## supplementary crystallographic information

### Comment

The description of the structure of the title compound is part of a series of papers on mononuclear complexes of the type [Ln(C_12_H_8_N_2_)_2_(C_7_H_8_O_3_)_2_ (NO_3_)], with Ln = Ce, Pr, Sm (this publication), Eu, and Dy. All five compounds are isostructural to the previously reported Nd complex (Zheng *et al.* 2010). The background to this study is given in the previous paper by Zheng *et al*. (2010).

### Experimental

Each reagent was commercially available and of analytical grade. Sm(NO_3_)_3_.6H_2_O (0.222 g, 0.5 mmol), 2, 6-dihydroxybenzoic acid (0.074 g 0.5mmol), 1, 10-phenanthroline (0.090 g, 0.5 mmol) and NaHCO_3_ (0.042 g, 0.5 mmol) were dissolved in water-ethanol solution (10 ml, 5:5). The solution was refluxed for 4 h, and filtered after cooling to room temperature. Orange single crystals were obtained from the filtrate after 3 days.

### Refinement

H atoms were positioned geometrically (C—H = 0.93 Å and O—H = 0.82 Å) and refined as riding, with *U*_iso _(H) = 1.2Ueq (C) and *U*_iso_(H) = 1.5Ueq (O).

### Crystal data

**Table:**

[Sm(C_7_H_5_O_3_)_2_(NO_3_)(C_12_H_8_N_2_)_2_]	*F*(000) = 1756
*M**_r_* = 878.99	*D*_x_ = 1.715 Mg m^−^^3^
Monoclinic, *P*2_1_/*c*	Cu *K*α radiation, λ = 1.54184 Å
Hall symbol: -P 2ybc	Cell parameters from 7456 reflections
*a* = 11.2022 (3) Å	θ = 3.3–67.5°
*b* = 26.7672 (7) Å	µ = 13.59 mm^−^^1^
*c* = 14.3326 (5) Å	*T* = 298 K
β = 127.635 (2)°	Prism, orange
*V* = 3403.4 (2) Å^3^	0.40 × 0.35 × 0.33 mm
*Z* = 4	

### Data collection

**Table:**

Oxford Diffraction Gemini S Ultra diffractometer	6027 independent reflections
Radiation source: Enhance Ultra (Cu) X-ray Source	5456 reflections with *I* > 2σ(*I*)
mirror	*R*_int_ = 0.045
Detector resolution: 15.9149 pixels mm^-1^	θ_max_ = 67.5°, θ_min_ = 3.3°
ω scans	*h* = −12→13
Absorption correction: multi-scan [*ABSPACK* in *CrysAlis PRO RED* (Oxford Diffraction, 2006)]	*k* = −31→30
*T*_min_ = 0.074, *T*_max_ = 0.094	*l* = −17→17
11856 measured reflections	

### Refinement

**Table:**

Refinement on *F*^2^	Primary atom site location: structure-invariant direct methods
Least-squares matrix: full	Secondary atom site location: difference Fourier map
*R*[*F*^2^ > 2σ(*F*^2^)] = 0.048	Hydrogen site location: inferred from neighbouring sites
*wR*(*F*^2^) = 0.122	H-atom parameters constrained
*S* = 1.06	*w* = 1/[σ^2^(*F*_o_^2^) + (0.0765*P*)^2^] where *P* = (*F*_o_^2^ + 2*F*_c_^2^)/3
6027 reflections	(Δ/σ)_max_ = 0.001
496 parameters	Δρ_max_ = 1.36 e Å^−^^3^
0 restraints	Δρ_min_ = −2.76 e Å^−^^3^
0 constraints	

### Special details

**Table:**

Geometry. All e.s.d.'s (except the e.s.d. in the dihedral angle between two l.s. planes) are estimated using the full covariance matrix. The cell e.s.d.'s are taken into account individually in the estimation of e.s.d.'s in distances, angles and torsion angles; correlations between e.s.d.'s in cell parameters are only used when they are defined by crystal symmetry. An approximate (isotropic) treatment of cell e.s.d.'s is used for estimating e.s.d.'s involving l.s. planes.
Refinement. Refinement of *F*^2^ against ALL reflections. The weighted *R*-factor *wR* and goodness of fit *S* are based on *F*^2^, conventional *R*-factors *R* are based on *F*, with *F* set to zero for negative *F*^2^. The threshold expression of *F*^2^ > σ(*F*^2^) is used only for calculating *R*-factors(gt) *etc*. and is not relevant to the choice of reflections for refinement. *R*-factors based on *F*^2^ are statistically about twice as large as those based on *F*, and *R*- factors based on ALL data will be even larger.

### Fractional atomic coordinates and isotropic or equivalent isotropic displacement parameters (Å^2^)

**Table:**

	*x*	*y*	*z*	*U*_iso_*/*U*_eq_	
Sm1	0.56897 (3)	0.861766 (8)	0.279589 (19)	0.02143 (11)	
O1	0.8344 (4)	0.89268 (14)	0.4021 (4)	0.0424 (9)	
O2	0.8043 (4)	0.81199 (13)	0.3668 (3)	0.0345 (8)	
O3	1.0803 (5)	0.93558 (17)	0.4708 (5)	0.0656 (14)	
H27	0.9948	0.9328	0.4511	0.098*	
O4	1.0183 (4)	0.75825 (14)	0.3973 (3)	0.0421 (9)	
H31	0.9363	0.7639	0.3823	0.063*	
O5	0.6113 (4)	0.91857 (13)	0.4429 (3)	0.0370 (8)	
O6	0.6545 (4)	0.83811 (13)	0.4803 (3)	0.0361 (8)	
O7	0.6655 (7)	0.97939 (15)	0.6052 (4)	0.0731 (17)	
H34	0.6395	0.9708	0.5403	0.110*	
O8	0.7432 (5)	0.80232 (14)	0.6803 (4)	0.0513 (11)	
H38	0.7138	0.8017	0.6121	0.077*	
O9	0.5441 (4)	0.80040 (13)	0.1275 (3)	0.0399 (8)	
O10	0.6521 (5)	0.87136 (14)	0.1517 (4)	0.0423 (9)	
O11	0.6352 (6)	0.8162 (2)	0.0333 (4)	0.0745 (16)	
N1	0.5255 (5)	0.95464 (14)	0.2095 (3)	0.0285 (8)	
N2	0.3339 (5)	0.88100 (14)	0.0607 (3)	0.0257 (8)	
N3	0.3170 (5)	0.86703 (14)	0.2519 (4)	0.0265 (8)	
N4	0.4413 (4)	0.77832 (13)	0.2615 (3)	0.0256 (8)	
N5	0.6111 (5)	0.82878 (18)	0.1017 (4)	0.0369 (10)	
C1	0.6194 (7)	0.99111 (19)	0.2810 (5)	0.0377 (12)	
H1	0.6950	0.9837	0.3598	0.045*	
C2	0.6085 (8)	1.0397 (2)	0.2421 (6)	0.0472 (14)	
H2	0.6764	1.0639	0.2947	0.057*	
C3	0.4987 (7)	1.05217 (17)	0.1271 (6)	0.0413 (13)	
H3	0.4911	1.0846	0.1008	0.050*	
C4	0.3974 (6)	1.01504 (17)	0.0494 (5)	0.0319 (10)	
C5	0.4155 (5)	0.96681 (16)	0.0939 (4)	0.0257 (9)	
C6	0.3146 (5)	0.92780 (17)	0.0160 (4)	0.0253 (9)	
C7	0.1982 (5)	0.9392 (2)	−0.1042 (4)	0.0323 (10)	
C8	0.1840 (6)	0.9888 (2)	−0.1461 (5)	0.0418 (13)	
H8	0.1082	0.9962	−0.2249	0.050*	
C9	0.2792 (7)	1.0252 (2)	−0.0727 (5)	0.0419 (13)	
H9	0.2681	1.0573	−0.1017	0.050*	
C10	0.1017 (6)	0.9002 (2)	−0.1772 (5)	0.0402 (12)	
H10	0.0255	0.9063	−0.2567	0.048*	
C11	0.1190 (6)	0.8536 (2)	−0.1323 (5)	0.0386 (12)	
H11	0.0546	0.8277	−0.1796	0.046*	
C12	0.2376 (6)	0.84599 (19)	−0.0121 (4)	0.0326 (11)	
H12	0.2491	0.8142	0.0185	0.039*	
C13	0.2574 (6)	0.9104 (2)	0.2502 (5)	0.0392 (12)	
H13	0.3166	0.9390	0.2751	0.047*	
C14	0.1097 (7)	0.9147 (2)	0.2125 (5)	0.0482 (15)	
H14	0.0715	0.9456	0.2119	0.058*	
C15	0.0213 (7)	0.8725 (3)	0.1762 (5)	0.0469 (14)	
H15	−0.0779	0.8749	0.1495	0.056*	
C16	0.0812 (6)	0.8262 (2)	0.1797 (4)	0.0359 (11)	
C17	0.2304 (5)	0.82545 (19)	0.2191 (4)	0.0295 (10)	
C18	0.2987 (5)	0.77830 (17)	0.2286 (4)	0.0267 (9)	
C19	0.2179 (6)	0.7335 (2)	0.2043 (4)	0.0340 (11)	
C20	0.0639 (7)	0.7363 (2)	0.1606 (5)	0.0438 (13)	
H20	0.0086	0.7070	0.1410	0.053*	
C21	−0.0021 (6)	0.7802 (3)	0.1475 (5)	0.0477 (15)	
H21	−0.1028	0.7809	0.1173	0.057*	
C22	0.2936 (7)	0.68849 (19)	0.2237 (5)	0.0400 (12)	
H22	0.2451	0.6582	0.2109	0.048*	
C23	0.4378 (7)	0.68904 (19)	0.2612 (4)	0.0385 (12)	
H23	0.4896	0.6593	0.2762	0.046*	
C24	0.5073 (6)	0.73497 (18)	0.2768 (4)	0.0344 (11)	
H24	0.6046	0.7350	0.2991	0.041*	
C25	0.8869 (6)	0.85025 (18)	0.4003 (4)	0.0295 (10)	
C26	1.0401 (6)	0.8472 (2)	0.4337 (4)	0.0315 (10)	
C27	1.1307 (6)	0.8902 (2)	0.4672 (5)	0.0403 (12)	
C28	1.2734 (7)	0.8871 (3)	0.4977 (5)	0.0538 (17)	
H28	1.3333	0.9154	0.5209	0.065*	
C29	1.3266 (6)	0.8405 (3)	0.4933 (5)	0.0505 (16)	
H29	1.4227	0.8382	0.5136	0.061*	
C30	1.2411 (6)	0.7983 (2)	0.4599 (5)	0.0406 (12)	
H30	1.2786	0.7678	0.4568	0.049*	
C31	1.0983 (6)	0.8009 (2)	0.4306 (4)	0.0331 (11)	
C32	0.6540 (5)	0.88185 (17)	0.5142 (4)	0.0278 (10)	
C33	0.7025 (5)	0.89010 (18)	0.6348 (4)	0.0274 (9)	
C34	0.7070 (7)	0.9386 (2)	0.6748 (5)	0.0408 (13)	
C35	0.7551 (9)	0.9461 (2)	0.7888 (6)	0.0562 (17)	
H35	0.7586	0.9782	0.8153	0.067*	
C36	0.7975 (7)	0.9058 (3)	0.8622 (5)	0.0512 (15)	
H36	0.8302	0.9111	0.9387	0.061*	
C37	0.7929 (7)	0.8582 (2)	0.8260 (5)	0.0438 (14)	
H37	0.8215	0.8315	0.8772	0.053*	
C38	0.7453 (6)	0.8496 (2)	0.7118 (4)	0.0321 (10)	

### Atomic displacement parameters (Å^2^)

**Table:**

	*U*^11^	*U*^22^	*U*^33^	*U*^12^	*U*^13^	*U*^23^
Sm1	0.01716 (16)	0.02004 (15)	0.02685 (16)	−0.00046 (8)	0.01331 (12)	0.00015 (8)
O1	0.0257 (19)	0.0378 (19)	0.059 (2)	−0.0055 (16)	0.0234 (18)	−0.0091 (17)
O2	0.0195 (17)	0.0341 (17)	0.0463 (19)	−0.0009 (14)	0.0183 (16)	0.0010 (15)
O3	0.042 (3)	0.049 (2)	0.090 (4)	−0.019 (2)	0.032 (3)	−0.017 (2)
O4	0.0255 (19)	0.0411 (19)	0.056 (2)	0.0000 (16)	0.0232 (18)	−0.0066 (17)
O5	0.039 (2)	0.0383 (19)	0.0333 (17)	0.0040 (16)	0.0219 (17)	0.0025 (15)
O6	0.035 (2)	0.0334 (18)	0.0323 (17)	−0.0015 (15)	0.0166 (16)	−0.0043 (14)
O7	0.132 (5)	0.033 (2)	0.048 (2)	0.018 (3)	0.052 (3)	0.0044 (18)
O8	0.056 (3)	0.0343 (19)	0.042 (2)	0.0019 (19)	0.019 (2)	0.0073 (16)
O9	0.039 (2)	0.0376 (18)	0.046 (2)	0.0016 (17)	0.0270 (18)	−0.0026 (16)
O10	0.046 (2)	0.0413 (19)	0.051 (2)	−0.0043 (18)	0.036 (2)	0.0058 (17)
O11	0.070 (3)	0.124 (5)	0.049 (2)	0.007 (3)	0.046 (3)	−0.013 (3)
N1	0.029 (2)	0.0232 (18)	0.0339 (19)	−0.0011 (16)	0.0193 (18)	−0.0021 (15)
N2	0.024 (2)	0.0254 (18)	0.0298 (19)	0.0005 (16)	0.0177 (17)	0.0009 (15)
N3	0.021 (2)	0.033 (2)	0.0279 (19)	0.0067 (16)	0.0162 (18)	0.0055 (15)
N4	0.020 (2)	0.0229 (18)	0.0308 (19)	−0.0014 (15)	0.0141 (16)	0.0030 (15)
N5	0.028 (2)	0.052 (3)	0.031 (2)	0.010 (2)	0.0185 (19)	0.0044 (19)
C1	0.043 (3)	0.030 (2)	0.046 (3)	−0.010 (2)	0.031 (3)	−0.009 (2)
C2	0.055 (4)	0.033 (3)	0.065 (4)	−0.014 (3)	0.042 (3)	−0.013 (3)
C3	0.059 (4)	0.017 (2)	0.068 (4)	0.002 (2)	0.049 (3)	0.001 (2)
C4	0.038 (3)	0.024 (2)	0.051 (3)	0.010 (2)	0.036 (3)	0.009 (2)
C5	0.025 (2)	0.023 (2)	0.039 (2)	0.0066 (18)	0.024 (2)	0.0037 (18)
C6	0.020 (2)	0.029 (2)	0.033 (2)	0.0073 (18)	0.020 (2)	0.0052 (18)
C7	0.020 (2)	0.043 (3)	0.034 (2)	0.009 (2)	0.017 (2)	0.008 (2)
C8	0.032 (3)	0.050 (3)	0.047 (3)	0.021 (3)	0.026 (3)	0.022 (3)
C9	0.045 (3)	0.034 (3)	0.061 (3)	0.021 (2)	0.040 (3)	0.023 (2)
C10	0.023 (3)	0.060 (3)	0.033 (3)	0.004 (2)	0.014 (2)	0.003 (2)
C11	0.018 (3)	0.056 (3)	0.031 (3)	−0.006 (2)	0.009 (2)	−0.006 (2)
C12	0.032 (3)	0.030 (2)	0.034 (2)	−0.004 (2)	0.020 (2)	−0.001 (2)
C13	0.035 (3)	0.040 (3)	0.045 (3)	0.010 (2)	0.025 (3)	0.001 (2)
C14	0.038 (3)	0.058 (3)	0.048 (3)	0.028 (3)	0.026 (3)	0.013 (3)
C15	0.024 (3)	0.076 (4)	0.041 (3)	0.017 (3)	0.020 (3)	0.013 (3)
C16	0.022 (2)	0.057 (3)	0.027 (2)	0.004 (2)	0.014 (2)	0.005 (2)
C17	0.023 (2)	0.041 (3)	0.023 (2)	0.000 (2)	0.0140 (19)	0.0029 (19)
C18	0.024 (2)	0.033 (2)	0.024 (2)	−0.0042 (19)	0.0152 (19)	−0.0009 (17)
C19	0.033 (3)	0.044 (3)	0.023 (2)	−0.017 (2)	0.015 (2)	−0.0051 (19)
C20	0.034 (3)	0.057 (3)	0.039 (3)	−0.021 (3)	0.022 (2)	−0.005 (3)
C21	0.023 (3)	0.082 (4)	0.035 (3)	−0.013 (3)	0.016 (2)	0.002 (3)
C22	0.052 (4)	0.030 (2)	0.038 (3)	−0.013 (2)	0.028 (3)	−0.002 (2)
C23	0.048 (3)	0.028 (2)	0.036 (3)	0.001 (2)	0.023 (2)	0.004 (2)
C24	0.032 (3)	0.029 (2)	0.042 (3)	0.004 (2)	0.023 (2)	0.002 (2)
C25	0.021 (2)	0.032 (2)	0.029 (2)	−0.002 (2)	0.012 (2)	−0.0017 (19)
C26	0.018 (2)	0.041 (3)	0.026 (2)	−0.005 (2)	0.0085 (19)	−0.002 (2)
C27	0.027 (3)	0.050 (3)	0.035 (3)	−0.011 (2)	0.014 (2)	−0.005 (2)
C28	0.035 (3)	0.074 (4)	0.044 (3)	−0.026 (3)	0.020 (3)	−0.005 (3)
C29	0.022 (3)	0.090 (5)	0.036 (3)	−0.008 (3)	0.015 (2)	0.001 (3)
C30	0.021 (3)	0.067 (4)	0.033 (2)	0.005 (2)	0.016 (2)	0.000 (2)
C31	0.023 (2)	0.052 (3)	0.024 (2)	0.000 (2)	0.014 (2)	0.001 (2)
C32	0.019 (2)	0.031 (2)	0.030 (2)	−0.0010 (19)	0.014 (2)	0.0001 (19)
C33	0.017 (2)	0.035 (2)	0.027 (2)	−0.0003 (18)	0.0115 (19)	−0.0017 (18)
C34	0.047 (3)	0.037 (3)	0.034 (3)	0.012 (2)	0.022 (3)	0.003 (2)
C35	0.075 (5)	0.050 (3)	0.044 (3)	0.011 (3)	0.037 (3)	−0.006 (3)
C36	0.047 (4)	0.074 (4)	0.029 (3)	0.009 (3)	0.021 (3)	0.003 (3)
C37	0.029 (3)	0.062 (4)	0.030 (3)	0.004 (2)	0.013 (2)	0.010 (2)
C38	0.016 (2)	0.039 (2)	0.030 (2)	−0.003 (2)	0.009 (2)	0.003 (2)

### Geometric parameters (Å, °)

**Table:**

Sm1—O6	2.496 (4)	C9—H9	0.9300
Sm1—O1	2.497 (4)	C10—C11	1.363 (8)
Sm1—O2	2.503 (3)	C10—H10	0.9300
Sm1—O10	2.526 (4)	C11—C12	1.407 (7)
Sm1—N4	2.579 (4)	C11—H11	0.9300
Sm1—O5	2.581 (3)	C12—H12	0.9300
Sm1—N3	2.603 (4)	C13—C14	1.397 (8)
Sm1—O9	2.605 (4)	C13—H13	0.9300
Sm1—N1	2.613 (4)	C14—C15	1.377 (10)
Sm1—N2	2.637 (4)	C14—H14	0.9300
O1—C25	1.286 (6)	C15—C16	1.395 (8)
O2—C25	1.262 (6)	C15—H15	0.9300
O3—C27	1.353 (8)	C16—C17	1.400 (7)
O3—H27	0.8200	C16—C21	1.442 (8)
O4—C31	1.345 (6)	C17—C18	1.439 (7)
O4—H31	0.8200	C18—C19	1.413 (7)
O5—C32	1.281 (6)	C19—C22	1.399 (8)
O6—C32	1.269 (6)	C19—C20	1.433 (8)
O7—C34	1.355 (7)	C20—C21	1.338 (9)
O7—H34	0.8200	C20—H20	0.9300
O8—C38	1.338 (7)	C21—H21	0.9300
O8—H38	0.8200	C22—C23	1.355 (8)
O9—N5	1.271 (6)	C22—H22	0.9300
O10—N5	1.273 (6)	C23—C24	1.398 (7)
O11—N5	1.212 (6)	C23—H23	0.9300
N1—C1	1.339 (6)	C24—H24	0.9300
N1—C5	1.370 (6)	C25—C26	1.475 (7)
N2—C12	1.325 (6)	C26—C27	1.411 (7)
N2—C6	1.363 (6)	C26—C31	1.415 (8)
N3—C13	1.332 (6)	C27—C28	1.378 (9)
N3—C17	1.358 (6)	C28—C29	1.401 (11)
N4—C24	1.321 (6)	C28—H28	0.9300
N4—C18	1.361 (6)	C29—C30	1.365 (9)
C1—C2	1.391 (8)	C29—H29	0.9300
C1—H1	0.9300	C30—C31	1.386 (7)
C2—C3	1.366 (9)	C30—H30	0.9300
C2—H2	0.9300	C32—C33	1.479 (7)
C3—C4	1.405 (8)	C33—C38	1.406 (7)
C3—H3	0.9300	C33—C34	1.407 (7)
C4—C5	1.399 (6)	C34—C35	1.388 (8)
C4—C9	1.434 (8)	C35—C36	1.373 (9)
C5—C6	1.440 (7)	C35—H35	0.9300
C6—C7	1.420 (7)	C36—C37	1.367 (9)
C7—C10	1.403 (8)	C36—H36	0.9300
C7—C8	1.426 (7)	C37—C38	1.395 (8)
C8—C9	1.350 (9)	C37—H37	0.9300
C8—H8	0.9300		
			
O6—Sm1—O1	79.60 (13)	C9—C8—H8	119.6
O6—Sm1—O2	75.13 (12)	C7—C8—H8	119.6
O1—Sm1—O2	52.14 (11)	C8—C9—C4	121.0 (5)
O6—Sm1—O10	144.12 (14)	C8—C9—H9	119.5
O1—Sm1—O10	70.61 (14)	C4—C9—H9	119.5
O2—Sm1—O10	70.96 (13)	C11—C10—C7	120.4 (5)
O6—Sm1—N4	72.23 (12)	C11—C10—H10	119.8
O1—Sm1—N4	135.00 (12)	C7—C10—H10	119.8
O2—Sm1—N4	86.53 (11)	C10—C11—C12	117.8 (5)
O10—Sm1—N4	116.30 (13)	C10—C11—H11	121.1
O6—Sm1—O5	51.34 (11)	C12—C11—H11	121.1
O1—Sm1—O5	71.74 (13)	N2—C12—C11	124.6 (5)
O2—Sm1—O5	107.85 (12)	N2—C12—H12	117.7
O10—Sm1—O5	130.81 (12)	C11—C12—H12	117.7
N4—Sm1—O5	112.62 (12)	N3—C13—C14	122.7 (6)
O6—Sm1—N3	78.86 (13)	N3—C13—H13	118.6
O1—Sm1—N3	143.53 (13)	C14—C13—H13	118.6
O2—Sm1—N3	144.92 (11)	C15—C14—C13	119.2 (5)
O10—Sm1—N3	136.88 (13)	C15—C14—H14	120.4
N4—Sm1—N3	63.14 (12)	C13—C14—H14	120.4
O5—Sm1—N3	71.81 (13)	C14—C15—C16	119.6 (5)
O6—Sm1—O9	125.05 (11)	C14—C15—H15	120.2
O1—Sm1—O9	105.69 (13)	C16—C15—H15	120.2
O2—Sm1—O9	67.92 (12)	C15—C16—C17	117.3 (5)
O10—Sm1—O9	49.84 (12)	C15—C16—C21	123.0 (5)
N4—Sm1—O9	66.46 (12)	C17—C16—C21	119.7 (5)
O5—Sm1—O9	175.58 (12)	N3—C17—C16	123.5 (5)
N3—Sm1—O9	110.77 (12)	N3—C17—C18	117.4 (4)
O6—Sm1—N1	122.32 (12)	C16—C17—C18	119.1 (5)
O1—Sm1—N1	79.47 (13)	N4—C18—C19	121.7 (5)
O2—Sm1—N1	126.21 (12)	N4—C18—C17	118.4 (4)
O10—Sm1—N1	71.89 (13)	C19—C18—C17	119.9 (5)
N4—Sm1—N1	145.46 (12)	C22—C19—C18	117.5 (5)
O5—Sm1—N1	71.10 (12)	C22—C19—C20	123.7 (5)
N3—Sm1—N1	87.69 (12)	C18—C19—C20	118.8 (5)
O9—Sm1—N1	112.25 (12)	C21—C20—C19	121.5 (5)
O6—Sm1—N2	145.35 (13)	C21—C20—H20	119.2
O1—Sm1—N2	131.85 (13)	C19—C20—H20	119.2
O2—Sm1—N2	132.86 (12)	C20—C21—C16	120.8 (5)
O10—Sm1—N2	70.09 (13)	C20—C21—H21	119.6
N4—Sm1—N2	87.71 (12)	C16—C21—H21	119.6
O5—Sm1—N2	117.46 (12)	C23—C22—C19	120.0 (5)
N3—Sm1—N2	66.80 (12)	C23—C22—H22	120.0
O9—Sm1—N2	66.95 (12)	C19—C22—H22	120.0
N1—Sm1—N2	62.66 (12)	C22—C23—C24	119.1 (5)
C25—O1—Sm1	93.2 (3)	C22—C23—H23	120.5
C25—O2—Sm1	93.6 (3)	C24—C23—H23	120.5
C27—O3—H27	109.5	N4—C24—C23	123.0 (5)
C31—O4—H31	109.5	N4—C24—H24	118.5
C32—O5—Sm1	92.5 (3)	C23—C24—H24	118.5
C32—O6—Sm1	96.8 (3)	O2—C25—O1	119.1 (5)
C34—O7—H34	109.5	O2—C25—C26	121.1 (5)
C38—O8—H38	109.5	O1—C25—C26	119.8 (5)
N5—O9—Sm1	94.9 (3)	C27—C26—C31	118.5 (5)
N5—O10—Sm1	98.7 (3)	C27—C26—C25	121.2 (5)
C1—N1—C5	117.3 (4)	C31—C26—C25	120.3 (5)
C1—N1—Sm1	121.7 (3)	O3—C27—C28	118.2 (6)
C5—N1—Sm1	120.7 (3)	O3—C27—C26	121.0 (5)
C12—N2—C6	117.4 (4)	C28—C27—C26	120.8 (6)
C12—N2—Sm1	122.7 (3)	C27—C28—C29	118.9 (6)
C6—N2—Sm1	119.7 (3)	C27—C28—H28	120.5
C13—N3—C17	117.6 (5)	C29—C28—H28	120.5
C13—N3—Sm1	122.3 (4)	C30—C29—C28	121.8 (5)
C17—N3—Sm1	119.2 (3)	C30—C29—H29	119.1
C24—N4—C18	118.5 (4)	C28—C29—H29	119.1
C24—N4—Sm1	121.6 (3)	C29—C30—C31	119.8 (6)
C18—N4—Sm1	119.8 (3)	C29—C30—H30	120.1
O11—N5—O9	122.1 (5)	C31—C30—H30	120.1
O11—N5—O10	121.5 (5)	O4—C31—C30	117.4 (5)
O9—N5—O10	116.4 (4)	O4—C31—C26	122.4 (5)
N1—C1—C2	122.7 (5)	C30—C31—C26	120.2 (5)
N1—C1—H1	118.7	O6—C32—O5	119.3 (4)
C2—C1—H1	118.7	O6—C32—C33	120.0 (4)
C3—C2—C1	120.4 (5)	O5—C32—C33	120.6 (4)
C3—C2—H2	119.8	C38—C33—C34	118.8 (5)
C1—C2—H2	119.8	C38—C33—C32	120.5 (4)
C2—C3—C4	118.7 (5)	C34—C33—C32	120.6 (4)
C2—C3—H3	120.6	O7—C34—C35	117.5 (5)
C4—C3—H3	120.6	O7—C34—C33	122.2 (5)
C5—C4—C3	118.0 (5)	C35—C34—C33	120.3 (5)
C5—C4—C9	120.0 (5)	C36—C35—C34	119.5 (6)
C3—C4—C9	122.0 (5)	C36—C35—H35	120.2
N1—C5—C4	123.0 (5)	C34—C35—H35	120.2
N1—C5—C6	117.8 (4)	C37—C36—C35	121.8 (5)
C4—C5—C6	119.2 (4)	C37—C36—H36	119.1
N2—C6—C7	122.1 (4)	C35—C36—H36	119.1
N2—C6—C5	118.5 (4)	C36—C37—C38	119.9 (5)
C7—C6—C5	119.3 (4)	C36—C37—H37	120.0
C10—C7—C6	117.6 (5)	C38—C37—H37	120.0
C10—C7—C8	122.7 (5)	O8—C38—C37	117.8 (5)
C6—C7—C8	119.6 (5)	O8—C38—C33	122.5 (5)
C9—C8—C7	120.8 (5)	C37—C38—C33	119.7 (5)

### Hydrogen-bond geometry (Å, °)

**Table:**

*D*—H···*A*	*D*—H	H···*A*	*D*···*A*	*D*—H···*A*
O4—H31···O2	0.82	1.87	2.594 (5)	147
O3—H27···O1	0.82	1.83	2.562 (6)	149
O8—H38···O6	0.82	1.85	2.578 (6)	147
O7—H34···O5	0.82	1.86	2.589 (5)	147
